# The Prevention of Diabetic Cardiomyopathy by Non-Mitogenic Acidic Fibroblast Growth Factor Is Probably Mediated by the Suppression of Oxidative Stress and Damage

**DOI:** 10.1371/journal.pone.0082287

**Published:** 2013-12-09

**Authors:** Chi Zhang, Linbo Zhang, Shali Chen, Biao Feng, Xuemian Lu, Yang Bai, Guang Liang, Yi Tan, Minglong Shao, Melissa Skibba, Litai Jin, Xiaokun Li, Subrata Chakrabarti, Lu Cai

**Affiliations:** 1 Chinese-American Research Institute for Diabetic Complications, Wenzhou Medical University, Wenzhou, Zhejiang, China; 2 Ruian Center of the Chinese-American Research Institute for Diabetic Complications, The Third Affiliated Hospital of Wenzhou Medical University, Wenzhou, Zhejiang, China; 3 Department of Pathology, Western University, London, Ontario, Canada; 4 Department of Pharmaceutical Engineering, Jilin Agriculture University, Changchun, Jilin, China; 5 Department of Cardiac Surgery, The First Hospital of Jilin University, Changchun, Jilin, China; 6 School of Pharmacy, Wenzhou Medical University, Wenzhou, Zhejiang, China; 7 Departments of Pharmacology and Toxicology, University of Louisville, Louisville, Kentucky, United States of America; 8 Kosair Children's Hospital Research Institute, Department of Pediatrics, University of Louisville, Louisville, Kentucky, United States of America; University of Western Ontario, Canada

## Abstract

**Background:**

Emerging evidence showed the beneficial effect of acidic fibroblast growth factor (aFGF) on heart diseases. The present study investigated whether non-mitogenic aFGF (nm-aFGF) can prevent diabetic cardiomyopathy and the underlying mechanisms, if any.

**Methodology/Principal Findings:**

Type 1 diabetes was induced in mice by multiple intraperitoneal injections of low-dose streptozotocin. Hyperglycemic and age-matched control mice were treated with or without nm-aFGF at 10 µg/kg daily for 1 and 6 months. Blood pressure and cardiac function were assessed. Cardiac H9c2 cell, human microvascular endothelial cells, and rat cardiomyocytes were exposed to high glucose (25 mM) for mimicking an in vitro diabetic condition for mechanistic studies. Oxidative stress, DNA damage, cardiac hypertrophy and fibrosis were assessed by real-time qPCR, immunofluorescent staining, Western blotting, and pathological examination. Nm-aFGF significantly prevented diabetes-induced hypertension and cardiac dysfunction at 6 months. Mechanistic studies demonstrated that nm-aFGF showed the similar preventive effect as the native aFGF on high glucose-induced oxidative stress (increase generation of reactive oxygen species) and damage (cellular DNA oxidation), cell hypertrophy, and fibrotic response (increased mRNA expression of fibronectin) in three kinds of cells. These in vitro findings were recaptured by examining the heart of the diabetic mice with and without nm-aFGF.

**Conclusions:**

These results suggest that nm-aFGF can prevent diabetic cardiomyopathy, probably through attenuation of cardiac oxidative stress, hypertrophy, and fibrosis.

## Introduction

Diabetic cardiomyopathy was considered to be associated with oxidative stress and DNA damage which are regarded as main incentives to initiate ventricular remodeling, characterized by cardiac hypertrophy and fibrosis and heart dysfunction [1]–[3]. Therefore, attenuation of diabetes-induced oxidative damage and the subsequent cardiac hypertrophy and fibrosis are expected to exert beneficial effects and may be a potential novel therapeutic strategy for diabetic cardiomyopathy.

Fibroblast growth factor (FGF) is a super family including at least 23 members. As the earliest found, the acidic FGF (aFGF or FGF-1) and basic FGF (bFGF or FGF-2) are regarded as the representatives of the FGF family, which are characterized by a high affinity to heparin and play critical role in cell proliferation [4]–[6]. It is reported that aFGF is highly expressed in the heart and stimulates angiogenesis [7], [8]. Cardiac aFGF participates in heart development and stimulates both cardiomyocyte proliferation and subsequent capillary angiogenesis [9]–[11]. In addition, expression of aFGF by adult cardiomyocyte can maintain cell survival by induction of DNA synthesis and regulation of gene expression [12]. Some studies mentioned that intracoronary, intrapericardial, or myocardial administration of aFGF or bFGF into ischemic canine and porcine hearts significantly minimized infarct size and improved cardiac function [13]–[15].

Diabetes was found to impair the expression of endogenous FGFs [16]. Some studies reported that administration of aFGF greatly improved wound healing under diabetic condition [17]–[19]. To date, whether aFGF can offer beneficial effect in the heart under diabetic condition is still unknown. However, growing evidence demonstrates that as an analogue of aFGF, bFGF induces significant cardio-protective effect under various pathological conditions [8], [20]–[22]. It was reported that bFGF prevented lipopolysaccharide-induced cardiac apoptosis through suppression of iNOS pathway [20], [21]. bFGF also protected against myocardial dysfunction and infarction induced by ischemia/reperfusion [22]. Our in vivo studies also confirmed that bFGF induced protection from ischemia/reperfusion induced cardiac injury in streptozotocin (STZ) induced type 1 diabetic rats [8]. Furthermore, a recent study also mentioned that aFGF improved cardio-protective effect in ischemic myocardium with ultrasound-mediated cavitation of heparin modified microbubbles [15].

Therefore, the present study was to test the hypothesis that aFGF can have a similar cardiac protection from diabetes-induced oxidative damage and subsequently remodeling. Considering that native FGF has the potential tumorigenic ability due to its non-specific stimulation of cell growth, we have modified the native aFGF by gene engineering to generate a non-mitogenic aFGF (nm-aFGF) that only loses the potential to stimulate cell proliferation with all other functions compared to native aFGF [23], [24]. To these ends, we applied STZ-induced type 1 diabetic mouse model with chronic treatment of nm-aFGF at the dose of 10 µg/kg body weight, based on previous studies [23], [24]. In addition, we also used in vitro cultures of cardiomyocytes, cardiac and endothelial cell lines, in combination of using in vivo cardiac tissues treated with and without nm-aFGF to perform mechanistic studies. We found that chronic administration of nm-aFGF can indeed protect the diabetes-induced cardiac dysfunction, which may be attributed to the suppression of cardiac oxidative damage, hypertrophy, and fibrosis.

## Materials and Methods

### Ethics Statement

All experimental procedures for the animal usage were approved by the Institutional Animal Care and Use Committee of the University of Louisville, which is compliant with the Guide for the Care and Use of Laboratory Animals published by the US National Institutes of Health (NIH Publication No. 85–23, revised 1996). The protocol was approved by the Institutional Animal Care and Use Committee of the University of Louisville (IACUC #: 10155). All surgery was performed under sodium avertin anesthesia, and all efforts were made to minimize suffering.

### Cell culture

Cardiomyoblasts (H9C2 cell line) were maintained in Dulbecco's modified Eagle's medium (DMEM) with 10% heat-inactivated fetal bovine serum, penicillin 100 IU/ml, and streptomycin 10 µg/ml. Cells were grown and maintained in 60 mm cell culture dishes at 37°C in a 5% CO_2_ humidified incubator. Glucose incubation (25 mmol/L) was performed for 48 hours with 200 ng/ml nm-aFGF pretreatment of cells in morphometric analysis mRNA expression detection.

Dermal-derived human microvascular endothelial cells (HMVECs) were obtained from Lonza Walkersville (Walkersville, USA). The culture conditions have been previously described [25]. Briefly, HMVECs were grown in EBM-2 (Lonza Walkersville) containing 1‰ human epidermal growth factor, 0.4‰ hydrocortisone, 1‰ gentamicin, 10% fetal bovine serum, 1‰ vascular endothelial growth factor, 4‰ human bFGF, 1‰ long R3 insulin-like growth factor 1, and 1‰ ascorbic acid. In both EBM and EBM-2, the glucose concentration was 5 mmol/L. Cells were grown in 25 cm^2^ tissue culture flasks and maintained in a humidified atmosphere containing 5% CO_2_ at 37°C. Cells at 80% confluence were growth-arrested by incubation in serum-free medium overnight before incubation with glucose (25 mmol/L). All experiments were carried out after 48 hours of glucose incubation with or without 1 hour pretreatment of nm-aFGF (200 ng/ml).

Neonatal rat cardiomyocytes were isolated from newborn Harlan Sprague-Dawley rat heart ventricles, as described previously [26]. Isolated primary cardiomyocytes were plated onto 6-cm cell culture dishes (Primaria Tissue Culture Dish; Becton Dickinson) at a density of 3.0×10^4^ cells/cm^2^ and were maintained for 48 hours in Dulbecco's Modified Eagle's Medium-Ham's F-12 supplemented with 10% fetal bovine serum, 10 µg/ml transferrin, 10 µg/ml insulin, 10 ng/ml selenium, 50 units/ml penicillin, 50 µg/ml streptomycin, 2 mg/ml bovine serum albumin, 5 µg/ml linoleic acid, 3 mM pyruvic acid, 0.1 mM minimum essential medium (MEM) nonessential amino acids, 10% MEM vitamin, 0.1 mM bromodeoxyuridine, 100 µM l-ascorbic acid, and 30 mM HEPES (pH 7.1). The cells were serum starved overnight prior to all experiments. Unless otherwise stated, all chemicals were of reagent grade quality and were purchased from Sigma Chemical (Sigma, Oakville, ON, Canada). All experiments were carried out after 48 hours of 25 mmol/L glucose incubation with or without nm-aFGF (200 ng/ml) pretreatment for 1 hour.

### Cellular reactive oxygen species levels

Intracellular reactive oxygen species (ROS) generation was assessed using an intracellular ROS assay kit from Cell Biolabs (San Diego, CA, USA) according to the manufacturer's instructions.

### Immunofluorescence

HMVECs and H9C2 cells were plated on eight-chamber tissue culture slides and incubated for 48 hours with the presence of glucose (25 mmol/L) and nm-aFGF (200 ng/ml), and then these cells were fixed with ethanol for staining with 8-OHdG antibody (Santa Cruz Biotehnology). Goat IgG labeled with FITC (Vector Laboratories, Burlingame, CA) was used for detection of the fluorescence. Slides were mounted in Vectashield fluorescence mounting medium with 4,6-diamidino-2-phenylindole (DAPI; Vector Labora- tories) for nuclear staining. Microscopic observation was performed by an examiner unaware of the identity of the sample, using a Zeiss LSM 410 inverted laser scan microscope equipped with fluorescein, rhodamine, and DAPI filters (Carl Zeiss Canada, North York, ON, Canada)

### Morphometric analysis

Cell surface areas were determined to assess cellular hypertrophy. Cells were visualized with a Leica inverted microscope, and images were captured at ×20 magnification. Cell area was determined using Mocha Software (SPSS). Cell surface area was determined from 50 randomly selected cells per petri dish and expressed as micrometers squared.

### Animals

To keep consistence with our previous study [27], male FVB mice, 8–10 weeks of age, were purchased from the Jackson Laboratory (Bar Harbor, Maine) and housed in the University of Louisville Research Resources Center at 22°C with a 12-h light/dark cycle and free access to standard rodent chow and tap water. For induction of the type 1 diabetes, mice were injected intraperitoneally with multiple -STZ [Sigma-Aldich, St. Louis, MO, dissolved in 0.1 M sodium citrate (pH 4.5)] at 50 mg/kg body weight daily for 5 consecutive days while age-matched control mice were received multiple injections of the same volume of sodium citrate buffer. Five days after the last injection of STZ, mice with hyperglycemia (blood glucose levels ≥250 mg/dl) were defined as diabetic as described previously [27]. Both diabetic and non-diabetic mice were treated with or without nm-aFGF treatment. Nm-aFGF (produced by our group using gene engineering approach) was intraperitoneally given at a dose of 10 µg/kg body weight daily for 1 and 6 months.

### Non-invasive blood pressure

Blood pressure (BP) was measured by tail-cuff manometry using a CODATM non-invasive BP monitoring system (Kent Scientific Corporation, Torrington, CT) at each time point. Mice were kept warm on the heating pad to ensure sufficient blood flow to the tail. Mice were restrained in a plastic tube restrainer. Occlusion and volume-pressure recording cuffs were placed over the tail. Each mouse was allowed to adapt to the restrainer for 5 min prior to BP measurement. The BP was measured for 10 acclimation cycles followed by 20 measurement cycles. After three days of training for the BP measurement, formal measurements for the unanesthetized BP and heart rate (HR) were collected (Table 1), as described previously [3].

**Table 1:** Effect of nm-aFGF on diabetes-induced unanesthetized blood pressure and heart rate.

	Control	nm-aFGF	DM	DM/nm-aFGF
**1 month**				
HR (beats/min)	667.71±46.63	647.97±59.62	644.1±29.21	589.62±49.98
Diastolic BP (mm Hg)	70.75±7.19	67.81±3.66	73.81±6. 42	71.44±5. 41
Systolic BP (mm Hg)	114.18±4.55	111.34±8.86	119.05±8.13	112.52±8.22
Mean BP (mm Hg)	94.52±5.46	89.63±2.791	90.42±4.57	91.82±8.29
**6 months**				
HR (beats/min)	645.45±23.45	641.76±39.79	654.1±35.15	639.62±44.88
Diastolic BP (mm Hg)	83.68±5.43	83.39±3.02	97.83±3.11*	73.44±8.33*^#^
Systolic BP (mm Hg)	113. 57±5.32	106. 48±5.62	129.14±8.34*	102.21±8.26*^#^
Mean BP (mm Hg)	94.25±5.61	89.63±4.18	104.42±4.03*	82.82±5.93*^#^

Notes: Data were presented as means±SEM. HR = heart rate; BP = blood pressure. *p<0.05 vs. control. # p<0.05 vs. DM group.

### Echocardiography

Transthoracic echocardiography (Echo) was performed for Avertin anesthetized mice at rest using a high-resolution imaging system for small animals (Vevo 770, VisualSonics, Canada), equipped with a high-frequent ultrasound probe (RMV-707B). All hair was removed from the chest using a chemical hair remover and the aquasonic clear ultrasound gel (Parker Laboratories, Fairfield, NJ) without bubbles and was applied to the thorax surface to optimize the visibility of the cardiac chambers. Parasternal long-axis and short-axis views were acquired. Left ventricular (LV) dimensions and wall thicknesses were determined from parasternal short axis M-mode images. The anesthetized HR was collected. Meanwhile, ejection fraction (EF), fractional shortening (FS), and LV mass were calculated by Vevo770 software (Table 2). The final data represent averaged values of 10 cardiac cycles [28].

**Table 2:** Effect of nm-aFGF on diabetes-induced cardiac dysfunction and anesthetized heart rate.

		Control	nm-aFGF	DM	DM/nm-aFGF
	HR (beats/min)	454±41.33	464.7±30.24	432.5±40.51	431.2±23.93
	LVID;d (mm)	3.31±0.09	3.34±0.11	3.37±0.12	3.29±0.14
	LVID;s (mm)	1.34±0.04	1.32±0.11	1.45±0.07	1.298±0.09
	IVS;d (mm)	0.72±0.01	0.71±0.02	0.72±0.01	0.73±0.03
	IVS;s (mm)	1.03±0.02	1.06±0.05	0.99±0.08	0.95±0.06
	LVPW;d (mm)	0.83±0.01	0.79±0.04	0.85±0.05	0.84±0.05
	LVPW;s (mm)	1.6±0.047	1.62±0.09	1.523±0.07	1.59±0.12
**1 month**	LV Vol;d (µL)	44.48±2.92	45.7±3.46	46.39±4.06	43.98±4.36
	LV Vol;s (µL)	4.62±0.38	4.57±0.76	6.51±0.73	4.28±0.75
	%EF (%)	89.69±0.14	90.15±1.65	87.98±1.32	89.95±0.85
	% FS (%)	59.61±1.12	60.49±2.39	56.97±1.84	59.72±1.23
	LV mass (mg)	82.49±3.76	81.08±4.05	83.13±6.76	83.09±9.52
	LVMC (mg)	65.99±3.01	64.87±3.24	66.5±5.41*	66.39±7.46
	HR (beats/min)	454.71±29.56	469.29±30.14	456.5±30.18	430.4±31.56
	LVID;d (mm)	3.55±0.24	3.43±0.19	4.52±0.54*	3.49±0.13*^#^
	LVID;s (mm)	1.55±0.06	1.61±0.05	2.55±0.08*	1.62±0.07*^#^
	IVS;d (mm)	0.77±0.05	0.78±0.04	0.92±0.04*	0.82±0.02*^#^
	IVS;s (mm)	1.05±0.03	1.07±0.05	0.82±0.03*	1.03±0.04*^#^
	LVPW;d (mm)	0.89±0.08	0.93±0.09	1.24±0.06*	0.96±0.06*^#^
**6 months**	LVPW;s (mm)	1.7±0.07	1.65±0.06	1.16±0.04*	1.64±0.08*^#^
	LV Vol;d (µL)	58.1±1.89	48.3±5.76	66.74±5.68*	50.7±4.36*^#^
	LV Vol;s (µL)	8.07±0.48	7.36±0.53	23.42±0.46*	7.41±0.36*^#^
	%EF (%)	86.11±4.68	85.29±5.54	67.02±5.96*	84.76±4.36*^#^
	% FS (%)	54.84±4.12	53.72±3.67	35.03±6.45*	52.87±4.34*^#^
	LV mass (mg)	114.8±5.28	100.76±7.91	146.45±8.1*	109.62±4.31*^#^
	LVMC (mg)	91.84±5.02	80.61±6.33	117.16±6.48*	87.7±3.45*^#^

Notes: Data were presented as means±SEM. LVID;d =  LV end-diastolic diameter; LVID;s =  LV end-systolic diameter; LVPW =  LV posterior wall; IVS =  interventricular septum; FS =  fractional shortening; EF =  ejection fraction; LVMC =  LV mass corrected. * p<0.05 vs. control. ^#^ p<0.05 vs. DM group.

### Sirius red staining of collagen

Tissue sections at 5 µm were used for Sirius Red staining of collagen with 0.1% Sirius Red F3BA and 0.25% Fast Green FCF. The sections stained for Sirius Red then were assessed for the proportion of fibrosis (collagen) using a computer-assisted image analysis system as described in our previous study [29].

### RNA extraction and real-time PCR

Trizol reagent (Invitrogen, Burlington, Canada) was used to isolate RNA as previously described [30]. RNA was extracted with chloroform followed by centrifugation to separate the sample into aqueous and organic phases. RNA was recovered from the aqueous phase by isopropyl alcohol precipitation and suspended in diethylpyrocarbonate-treated water. Total RNA (2 µg) was used for cDNA synthesis with high capacity cDNA reverse transcription kit (Applied Biosystems, Foster City, USA). The resulting cDNA products were stored at −20°C. Real-time RT-PCR was performed by using the LightCycler (Roche Diagnostics Canada, Laval, Canada), as previously described [31]. For a final reaction volume of 20 µL, the following reagents were added: 10 µL SYBR Advantage qPCR Premix (Clontech, Mountain View, USA), 1 µL each of forward and reverse 10 µmol/L primers, 7 µL H_2_O, and 1 µL cDNA template. The mRNA levels were quantified by using the standard curve method. Standard curves were constructed by using a serially diluted standard template. The data was normalized to 18S ribosomal RNA or β-actin RNA to account for differences in reverse transcription efficiencies and the amount of template in the reaction mixtures.

### Statistical analysis

Data is presented as mean ± standard error. Statistical significance of differences between groups was tested with Student's *t* test or one-way ANOVA followed by post hoc analysis, as appropriate. A p-value of 0.05 or less was considered to be significant. All calculations were performed with SPSS version 15.0 software.

## Results

### Chronic administration of nm-aFGF did not impact on diabetes-induced body weight loss and blood glucose increase, but significantly prevented diabetes-induced high blood pressure and cardiac dysfunction

**Figure 1:**
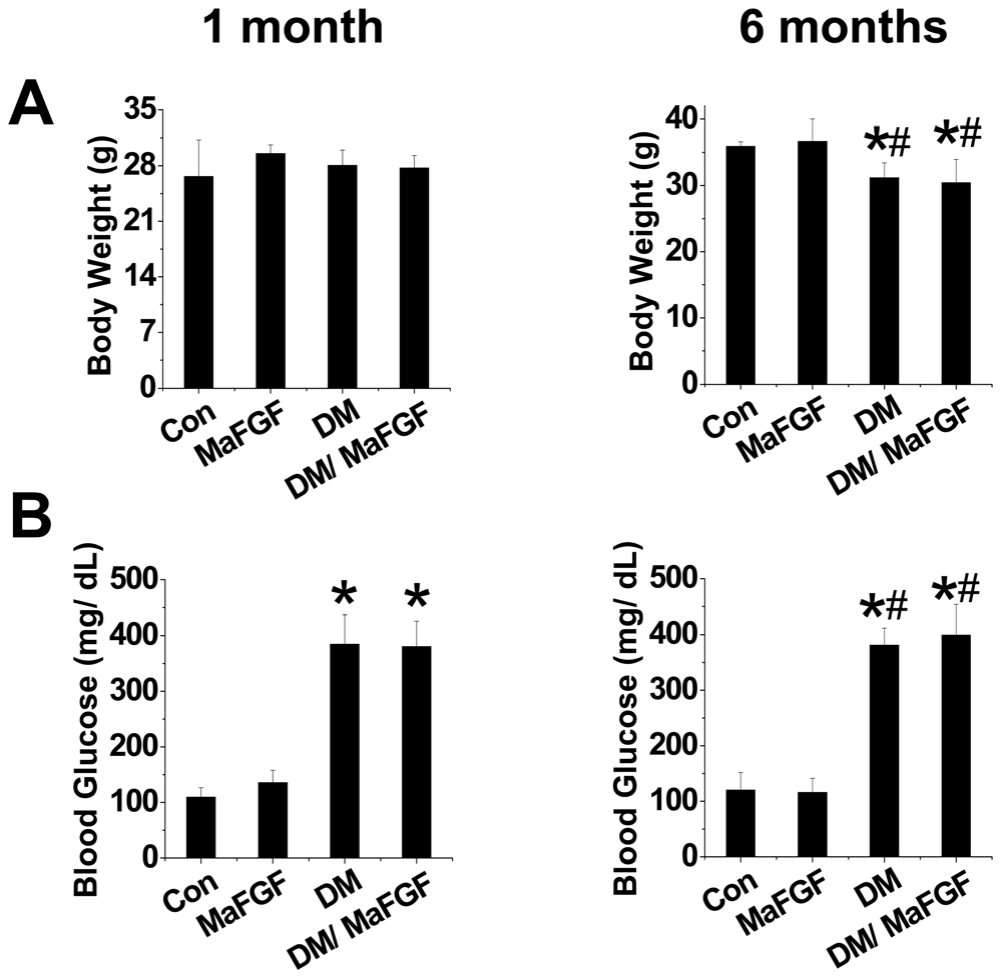
Effects of nm-aFGF on body weight, blood glucose levels in non-diabetic and diabetic mice. STZ induced type 1 diabetic FVB mice were intraperitoneally treated with or without nm-aFGF (10 µg/kg) treatment daily for either 1 or 6 months. The body weights (A) and blood glucose level (B) were monitored weekly and presented at indicated time points. Data are presented as means ± SD (n = 8). * p<0.05 vs control; # p<0.05 vs. DM (diabetic) group.

FVB mice were intraperitoneally injected with multiple low-dose STZs (50 mg/kg) to induce type 1 diabetes. Diabetic mice and age-matched non-diabetic mice were divided into groups with and without treatment of nm-aFGF (10 µg/kg daily) for 1 and 6 months. The body weight and blood glucose of the mice in each group were detected at each time point. Results showed that the body weight of the four groups were equal at 1 month after treatment (Fig. 1A). Meanwhile, blood glucose was significantly up-regulated in DM and DM/nm-aFGF groups (Fig. 1B). However, the body weight of DM mice greatly decreased at 6 months. Administration of nm-aFGF had no impact on the body weight loss and blood glucose levels (Fig. 1).

Unanesthetized BP and HR of these mice were examined by tail-cuff manometry. Diastolic, systolic, and averaged BPs were significantly increased at 6 months in DM group, which were not found at 1 month (Table 1). Chronic administration of nm-aFGF almost completely attenuated diabetes induced BPs increase. There was no significant difference of HR among groups at either 1 or 6 months (Table 1). The HRs of anesthetized mice, detected by Echo examination (Table 2), were lower than that of unanesthetized mice (Table 1), but were equal among groups at each time point. In addition, cardiac function was significantly impaired under diabetic condition at 6 months, rather than 1 month, with the characteristics of progressive increases in IVS;d, LVPW;d, LVID and LV Vol as well as progressive decreases in IVS;s, LVPW;s, %EF and %FS (Table 2). Administration of nm-aFGF almost completely prevented these cardiac dysfunctions in diabetic mice by reversing all the indices back to a normal level.

### In vitro mechanistic study on the preventive effect of nm-aFGF on high glucose induced oxidative stress, DNA oxidative damage, and the hypertrophic and fibrotic gene expression

#### Nm-aFGF prevented high glucose-induced oxidative stress and damage

**Figure 2:**
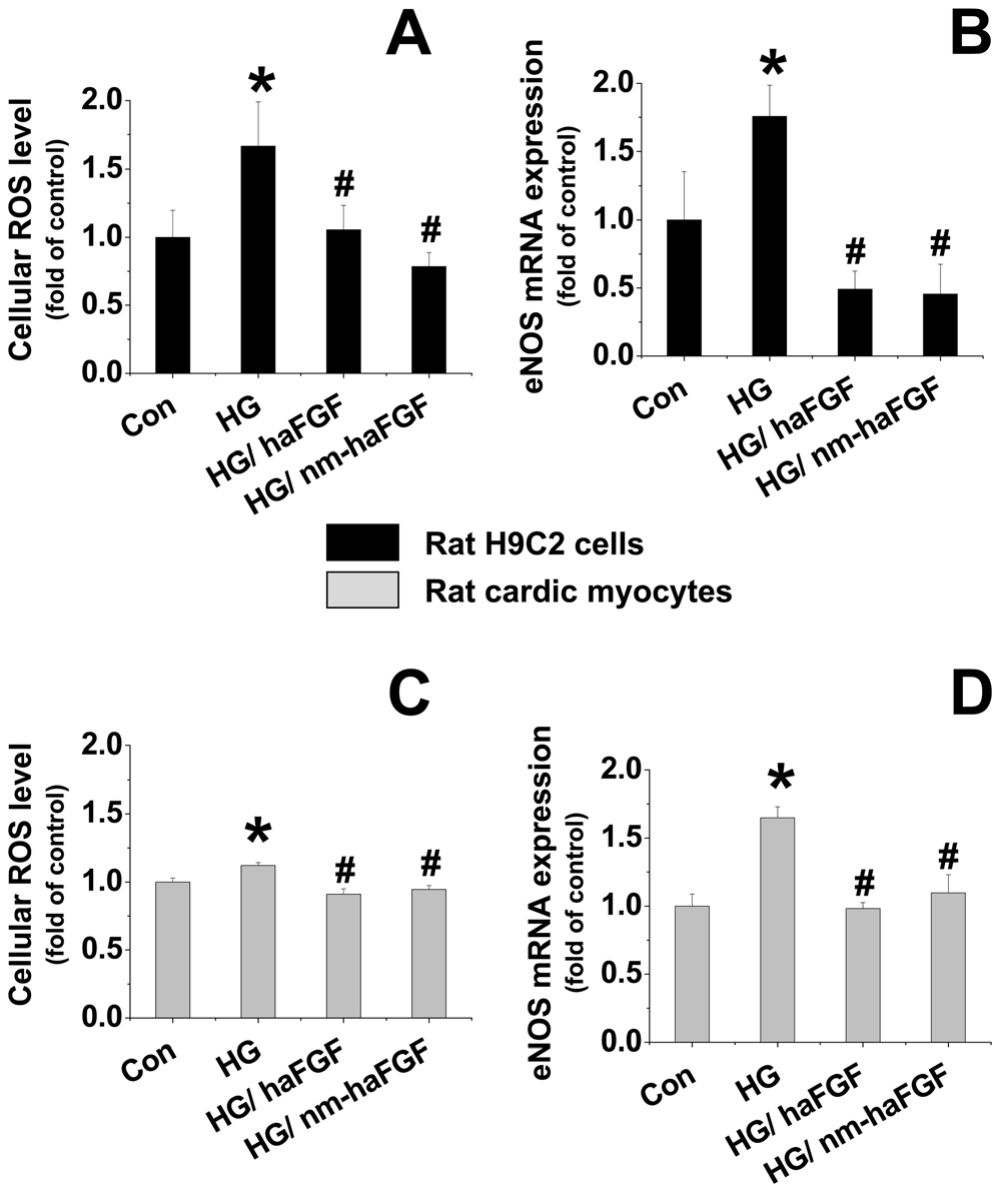
Effect of native aFGF or nm-aFGF on high glucose induced oxidative stress in cardiac cells. Pre- treatment of H9C2 cells and Rat cardiac myocytes with both native or nm-aFGF (200 ng/ml) for 1 hour followed by 48 hours treatment with both high glucose (25 mmol/L) and two types of aFGF. Both native aFGF and nm-aFGF prevented high glucose induced up-regulation of cellular ROS level (A) and eNOS mRNA expression (B) in either H9c2 or cardiac myocytes. Data were presented as means ± SD. All in vitro data were obtained from at least 3 independent experiments. * p<0.05 vs. control. # p<0.05 vs. high glucose (HG).

Strong evidence demonstrated that the development of diabetic cardiomyopathy is related to the induction of cardiac oxidative stress, characterized by production of ROS, leading to subsequent cell death, hypertrophy, and fibrosis. Therefore, we investigated whether the nm-aFGF induced cardiac protection by against diabetes was attributed to the suppression of oxidative stress. In our present study, we treated the H9c2 cells with high glucose (25 mmol/L) for 48 hours to mimic in vivo diabetic condition. Nm-aFGF was dissolved in PBS and added to a subset of cells with the working concentration of 200 ng/ml at 1 hour before and during high glucose treatment for 15 hours. We detected ROS level and the mRNA expression of endothelial nitric oxide synthase (eNOS) in both H9c2 cells and HMVECs. The results showed that high glucose increased cellular ROS level (Fig. 2A,C) and the mRNA expression of eNOS (Fig. 2B,D) in both H9c2 cells and HMVECs, which were significantly suppressed by both native and nm-aFGF treatments (Fig. 2) without significant difference between native aFGF-treated and nm-aFGF treated groups.

**Figure 3:**
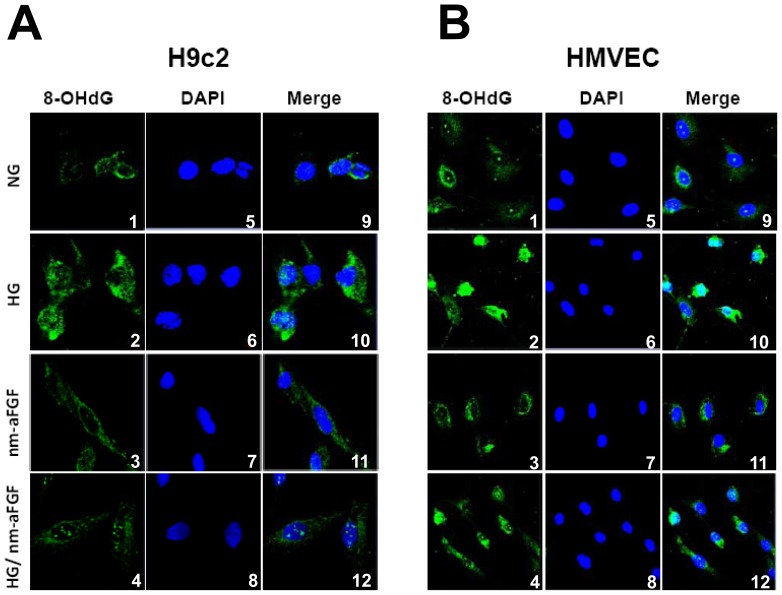
Effect of nm-aFGF on high glucose induced DNA oxidative damage in both H9c2 cells and HMVECs. Cells were exposed to nm-aFGF (200 ng/ml) for 1 hour prior to combination treatment of both high glucose (25 mmol/L) and nm-aFGF for 48 hours. After harvesting, cells were fixed for immunocytochemistry analysis of 8-OHdG (FITC A1-4, B1-4) and nuclear morphology (DAPI A5-8, B5-8) using fluorescence microscopy. DAPI staining is pseudocolored blue and 8-OHdG is shown in green. Images were merged and showed in A9-12 and B9-12. Each image is representative of at least 3 separate experiments.

Oxidative DNA damage was measured by immunofluorescent staining for 8-OHdG as the most sensitive approach. In line with the overgeneration of ROS (Fig. 2), the oxidative DNA damage positive staining increased in both cardiac H9c2 cells and HMVECs after 48-hour high glucose treatment (Fig. 3A,B, green), which was co-localized with that of nuclear dye DAPI (blue). The increased 8-OHdG stain in the nucleus induced by exposure to high glucose was significantly attenuated by treatment with nm-aFGF (200 ng/ml).

#### Nm-aFGF prevented high glucose-induced hypertrophic effect

**Figure 4:**
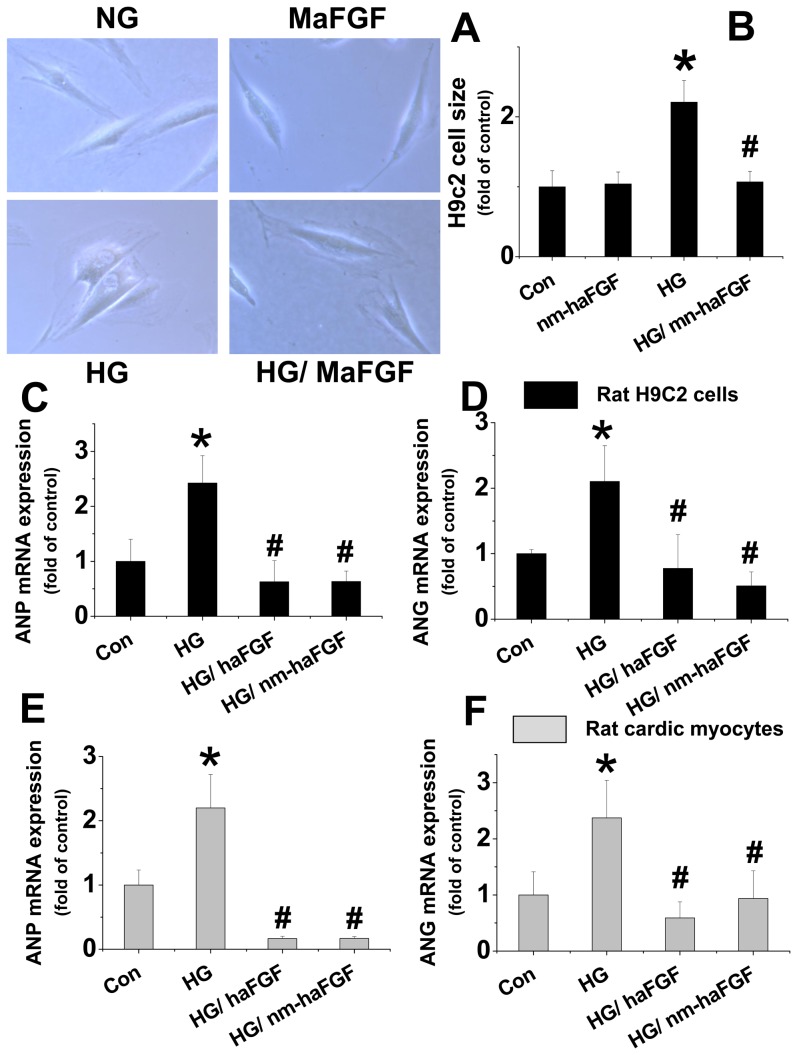
Both native aFGF and nm-aFGF showed preventive effect on high glucose induced cardiac cell hypertrophy in vitro. H9c2 cells and rat cardiomyocytes were treated as the same condition described in Figure 2. Cells were visualized with a Leica inverted microscope, and images were captured at ×20 magnification (A). Cell area was determined using Mocha software (B). Cell surface area was determined from 50 randomly selected cells per petri dish and expressed as micrometers squared. After harvesting, cardiac hypertrophic markers ANP mRNA (C, E) and ANG mRNA (D, F) expression in H9c2 and Rat cardiac myocytes were measured by real-time qPCR. Data were presented as means ± SD. All in vitro data were obtained from at least 3 independent experiments. * p<0.05 vs. control. # p<0.05 vs. high glucose (HG).

Oxidative stress has been considered one of the key contributors in the development of cardiac hypertrophy with the characteristic of cardiac cell size increase [32]–[34]. Therefore, the effect of nm-aFGF on cardiac cell size under high glucose conditions was investigated. We found that nm-aFGF had no impact on cell size under normal condition, but exposure to high glucose significantly increased the cell size (Fig. 4A), that was about 2 fold larger than the normal cell size (Fig. 4B). The cardiac cell hypertrophic effect induced by exposure to high glucose was almost completely prevented by nm-aFGF treatment.

To further explore the hypertrophic effect of cardiac cells induced by high glucose, we examined the mRNA expression of hypertrophic cytokines atrial natriuretic peptide (ANP) and angiotensinogen (ANG) [35]–[37]. High glucose significantly increased the mRNA expression of ANP and ANG in H9c2 cells (Fig. 4C,D) and rat cardiomyocytes (Fig. 4E,F), which was dramatically attenuated by native aFGF and nm-aFGF, respectively, without significant difference between two forms of FGFs.

#### Nm-aFGF prevented fibrotic response induced by high glucose

**Figure 5:**
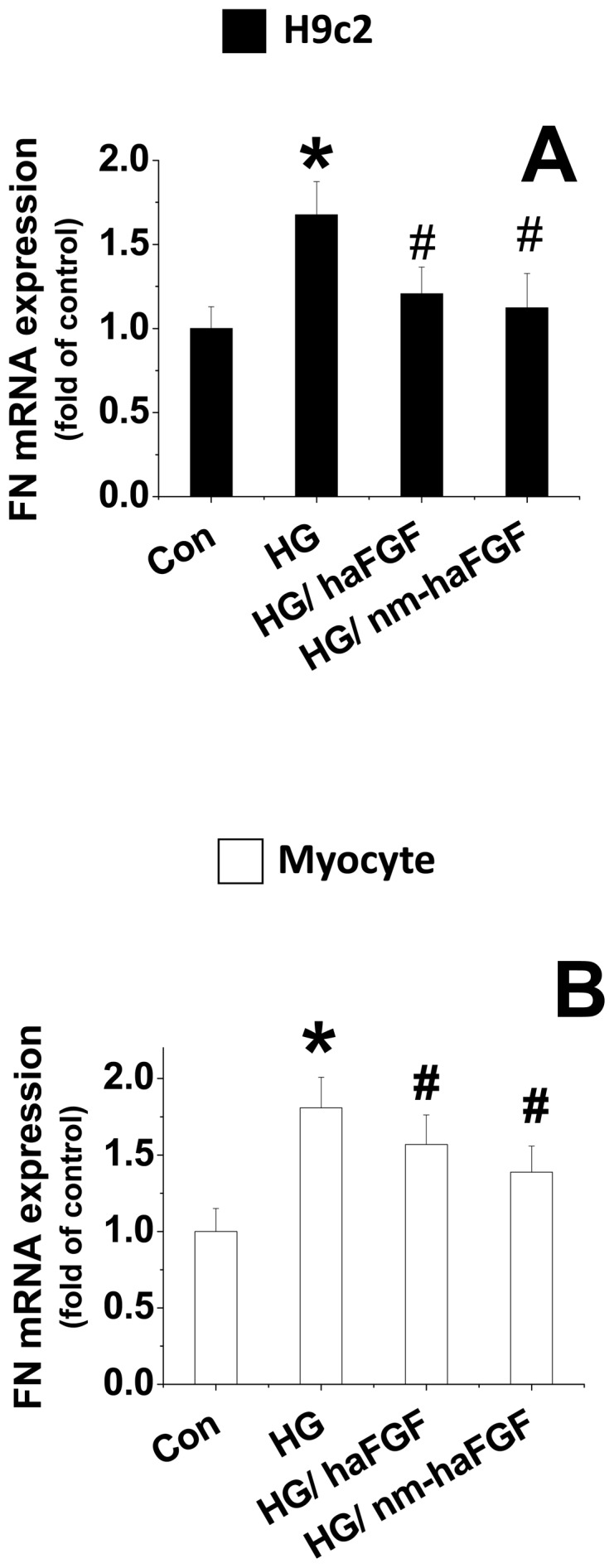
Both native aFGF and nm-aFGF showed preventive effect on high glucose induced mRNA expression upregulation of fibrotic markers in vitro. H9c2 cells, rat cardiomyocytes, and HMVECs were treated as described in Figure 2. After harvesting, fibrotic markers FN mRNA expression in H9c2 cells (A) and rat cardiomyocytes (B) were measured by real-time PCR. Data were presented as means ± SD. All in vitro data were obtained from at least 3 independent experiments. * p<0.05 vs. control. # p<0.05 vs. high glucose (HG).

Fibrosis plays a critical role in cardiac remodeling [38], [39]; therefore, we also examined fibrosis-related gene fibronectin (FN) expression under high glucose condition, with or without native/modified aFGF treatment in cardiac H9c2 cells and rat cardiomyocytes. We found FN mRNA expression was significantly upregulated after 48-hour exposure to high glucose (Fig. 5). Both native aFGF and nm-aFGF had significantly inhibitive effect on FN mRNA expression in H9c2 cells (Fig. 5A) and rat cardiomyocytes (Fig. 5B).

### Chronic administration of nm-aFGF prevented diabetes induced cardiac hypertrophy and fibrosis

**Figure 6:**
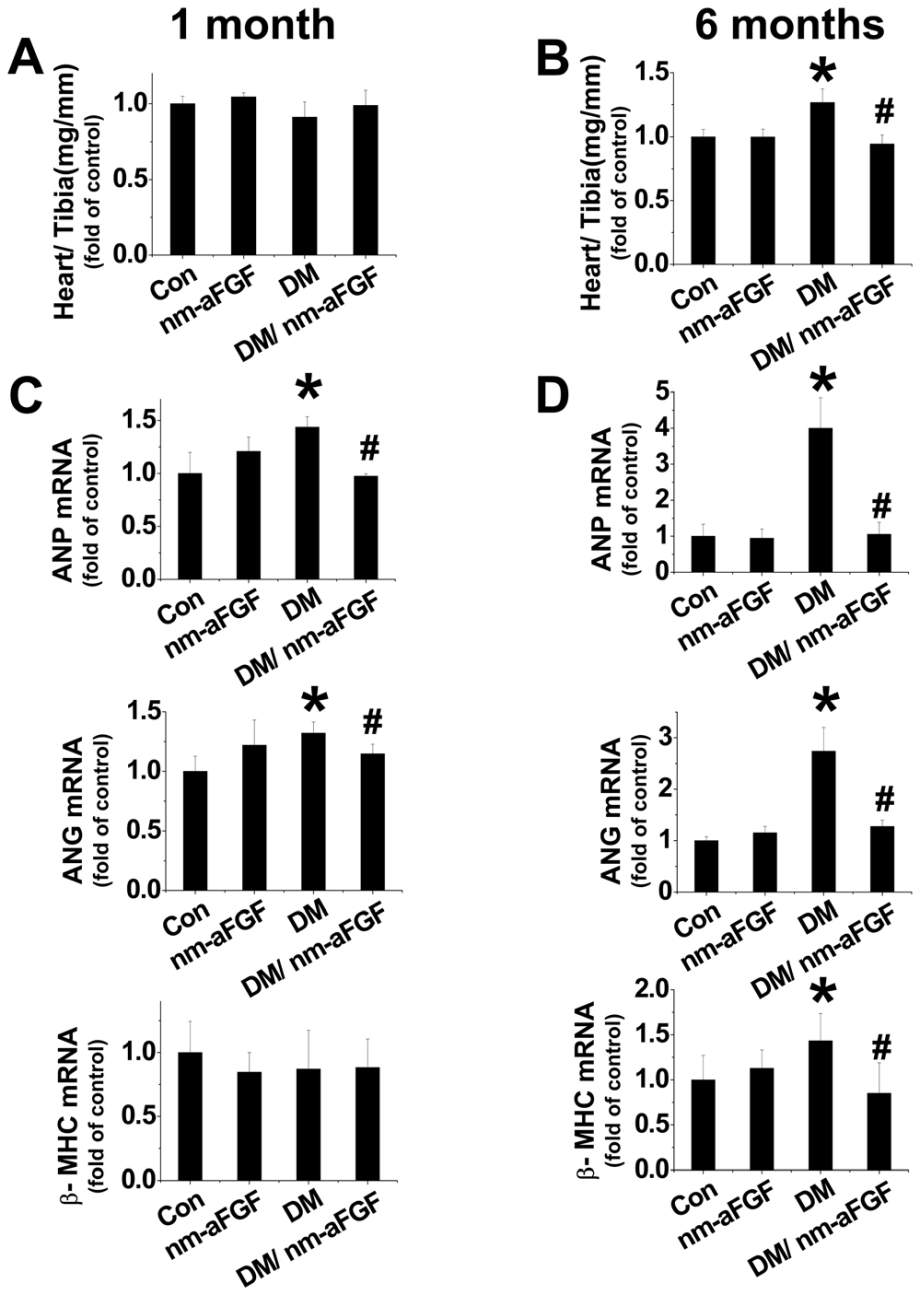
Prevention by nm-aFGF of diabetes-induced cardiac hypertrophy in vivo. Diabetic and age-matched mice were intraperitoneally injected with nm-aFGF at 10 µg/kg daily for 1 and 6 months. The ratio of the heart weight to tibia length (A, B) were measured and calculated after mice were sacrificed. Cardiac mRNA expression of the hypertrophic markers ANP mRNA (C, D), ANG mRNA (E, F) and β-MHC mRNA expression was measured by real-time qPCR. Data are presented as means ± SD (n = 8). * p<0.05 vs control; # p<0.05 vs DM (diabetic) group.

Our in vitro study revealed that nm-aFGF prevented high glucose-induced upregulation of ANP and ANG mRNA expression; therefore, we tested whether nm-aFGF plays a similar anti-hypertrophic role in vivo. Results from Table 2 showed that diabetes induced a significant increase of LV mass at 6 months, suggesting the possible induction of cardiac hypertrophy. This was confirmed by the ratio of the heart weight to tibia length of diabetic mice at 6 months (Fig. 6B) which was not found at 1 month (Fig. 6A).

Furthermore, molecular hypertrophy markers ANP, ANG and β-MHC were also significantly increased at mRNA level in the heart of diabetic mice 6 months after diabetes onset (Fig. 6D). Although diabetes-induced hypertrophy was not found at 1month according to the Echo data, the pathological changes already initiated at molecular level (Fig. 6C). Administration of nm-aFGF significantly prevented the hypertrophic changes at both 1 and 6 months (Table 2, Figs. 6).

**Figure 7:**
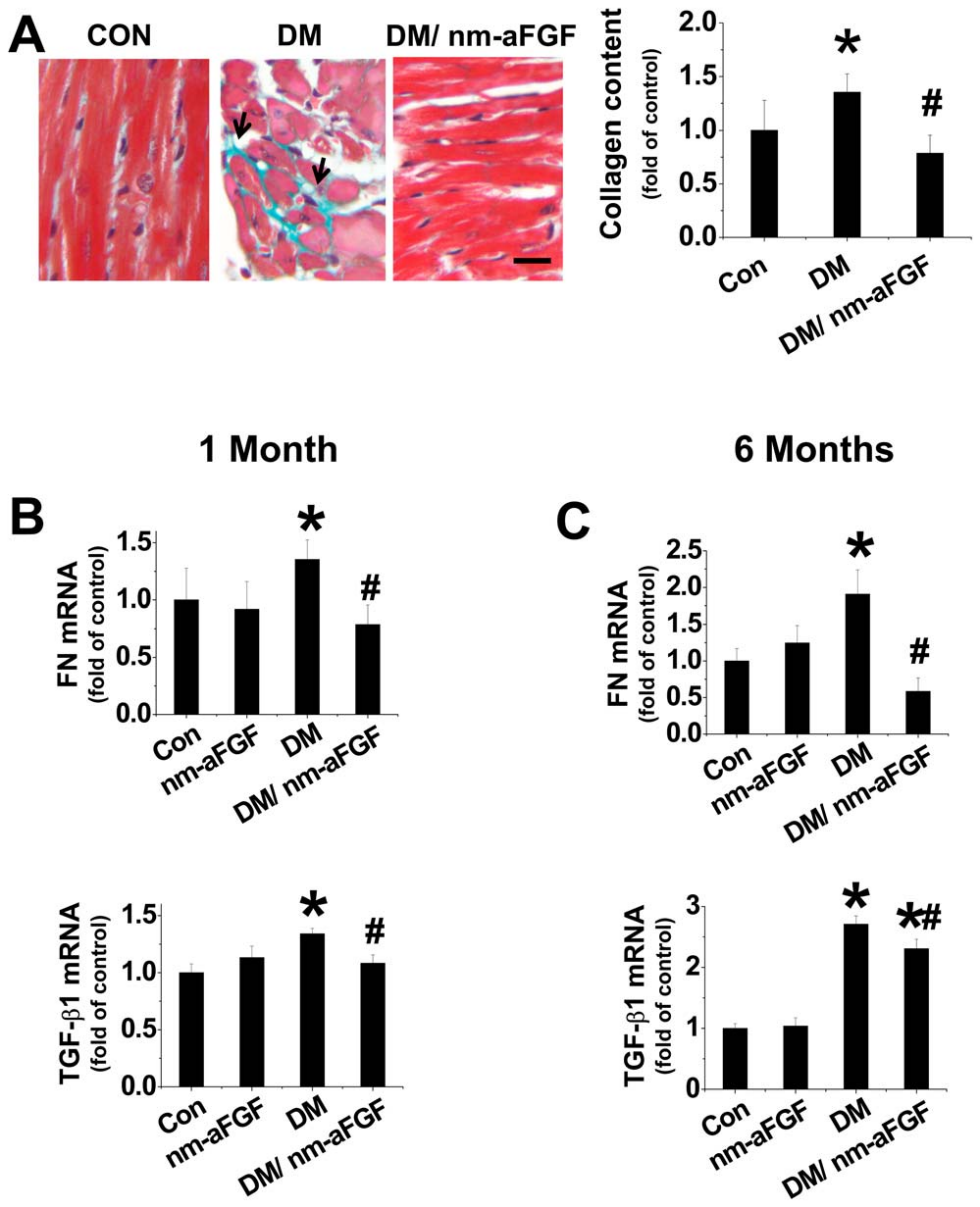
Prevention by nm-aFGF of diabetes-induced cardiac fibrosis in vivo. Mice were treated as described in Figure 6. Cardiac sections were subject to Sirius-red staining with 0.1% Sirius-red F3BA and 0.25% Fast green FCF for collagen accumulation and quantization by collagen content analysis (A). To further confirm the anti-fibrosis effect of nm-aFGF, the mRNA expression of fibrotic markers, including FN (B) and TGF-β1 (C) was measured by real-time PCR assay. Data are presented as means ± SD (n = 8). * p<0.05 vs control; # p<0.05 vs DM (diabetic) groups.

We also examined the fibrotic effect of diabetes on the heart by Sirius-red staining for collagen (Fig. 7A). Diabetes induced a significant amount of collagen accumulation, predominantly in interstitial, but also included the perivascular area, which was increased at 6 months. Nm-aFGF treatment completely prevented the collagen accumulation. DM-induced cardiac fibrosis was further confirmed by increased cardiac FN and TGF-β1 mRNA expressions (Fig. 7B, C) at both 1 and 6 months. Administration of nm-aFGF completely attenuated most of the fibrotic changes. For TGF-β1 mRNA at 6 months, although the therapeutic group was higher than normal mice, there was a slightly (but statistically different), decrease compared with non-treated diabetic group (Fig. 7).

## Discussion

Diabetes is a serious global issue nowadays, which leads to many kinds of fatal complications such as diabetic cardiomyopathy. Therefore, it is urgent to find a proper way to prevent oxidative stress and the consequently pathological changes in the heart.

We know that aFGF, highly expressed in the heart, is a specific well-defined angiogenic stimulant for the heart development. Cardiomyocyte-derived aFGF functions to increase the fetal ventricular cardiomyocyte population in absolute number as well as to facilitate the subsequent increase in capillary angiogenesis that occurs during cardiomyocyte maturation and ventricular remodeling [11]. As described above, the decrease of serum bFGF appears in infarction, stroke and peripheral vascular disease associated with diabetes [16]. Our previous study revealed that bFGF protected the heart in type 1 diabetes model [8]. We proposed that as an analogue of bFGF, aFGF may induce similar beneficial effect in the diabetic heart. In order to abrogate the oncogenicity of native aFGF, the nm-aFGF was applied in our present study to define whether nm-aFGF showed protection on diabetic cardiomyopathy.

In the present study using multiple-STZ-induced type 1 diabetic mouse model, the diabetic cardiomyopathy was successfully established at 6 months, shown by significantly cardiac dysfunction, including great increases in BPs, IVS;d, LVPW;d, LVID, LV Vol and LV mass along with significant decreases in LV EF and FS. We reported for the first time that chronic administration of nm-aFGF significantly prevented diabetes-induced hypertension and cardiac dysfunction at 6 months. It should be mentioned that there was no preventive effect of nm-aFGF on diabetic heart at the time of 1 month after diabetes since there was no significant manifestation of diabetic pathological changes.

From onset of diabetes to the development of diabetic cardiomyopathy, there are several pathological developing steps. Accumulated evidence indicates that oxidative stress may play a key role in the etiology of diabetic cardiomyopathy [40]. Under physiological conditions, ROS are continuously produced in cardiac cells, but the levels of ROS are regulated by a number of enzymes and antioxidants, so normally there is a physiological balance between ROS and antioxidants. However, under pathological conditions such as diabetes, hyperglycemia can destroy the balance with overproduction of ROS, leading to oxidative stress condition. As a result, oxidative stress will initiate harmful effect on cardiac cells, resulting in myocardial cell death and consequent hypertrophy and fibrosis, finally leading to diabetic dysfunction (cardiomyopathy) [41]. Some studies mentioned that the expression of aFGF was up-regulated by oxidative stress, implying that the increase of aFGF expression was an adaptive response and may show a protective effect against oxidative stress [42]–[44]. Since hyperglycemia is the predominant trigger of diabetic complications, the mechanistic studies here were performed with the H9c2 cells exposed to high glucose for mimicking diabetic hyperglycemia in vitro. For these studies we added native or modified aFGF into the medium of H9c2 cell culture to investigate whether aFGF can induce anti-oxidative effect in the H9c2 cells under high glucose conditions. The level of ROS and eNOS were examined in this study. We found that both ROS and eNOS levels were increased under high glucose condition, which were significantly attenuated by either native aFGF or nm-aFGF treatment. This implies that anti-oxidative function of aFGF might be the mechanism to prevent diabetic cardiac dysfunction and cardiomyopathy.

It is worthy to mention that nm-aFGF exhibited an equal anti-oxidation capacity to native aFGF even though nm-aFGF does not have mitogenic effect as we have demonstrated before [45], [46]. Therefore, nm-aFGF will have no oncogenic effect compared to the native aFGF, suggesting that nm-aFGF has more potential for clinical implications. In order to ensure the general protection by nm-aFGF from high glucose-induced oxidative stress in other cells, HMVECs were also applied in this study, which showed similar results: both native and modified aFGF significantly prevented high glucose-induced increases in cellular ROS level and cardiac eNOS mRNA expression.

Oxidative stress-induced cardiac cell death is attributed to DNA damage, including chromatin cross-linking, chromosome deletion, DNA strand breaks and base oxidation [47]. We know that aFGF plays a key role in the stimulation of cell proliferation and the prevention of cell death [45]. Cultured cells in the medium without FGF showed slow cell proliferation with cell death [48]. Therefore, in the present study we also tried to determine whether nm-aFGF could protect oxidative DNA damage of cardiac cells exposed to high glucose. Both H9c2 cells and HMVECs were treated as mentioned above, which showed that under normal conditions, the staining of oxidative DNA damage marker 8-OHDG was very low in both cell types. Treatment of normal cells with nm-aFGF had no impact on 8-OHDG staining; however, 8-OHDG staining was dramatically increased in the cells exposed to high glucose, but not in the cells exposed to both high glucose and nm-aFGF treatment. Therefore, nm-aFGF protects high glucose-induced oxidative DNA damage in cardiac cells.

Cardiac hypertrophy is characterized by enlargement of the heart caused by an increased myocyte size, which is generally associated with numerous side effects, including depressed LV ER%, heart failure and overall mortality [32], [49]. In term of its mechanism, a growing body of evidence revealed that oxidative stress plays a key role in the development of cardiac hypertrophy [32]. In cultured cardiomyocytes, hypertrophy induced by angiotensin II, endothelin 1, tumor necrosis factor-α or pulsatile mechanical stretch has been shown to be associated with the increase of intracellular ROS production [50]. Another study reported that ROS production contributed to the development of LV hypertrophy during chronic pressure overload [51]. The most widely recognized effect of increased oxidative stress is the oxidation and damage of macromolecules, membranes, DNA and enzymes involved in cellular function and homeostasis [52]. In the present study we demonstrated that nm-aFGF showed almost equal beneficial effects to the native aFGF on the prevention of high glucose-induced oxidative stress and oxidative DNA damage. Due to the close relationship between oxidative stress and cardiac cell hypertrophy, we also tried to identify whether nm-aFGF showed protection of high glucose-induced cardiac cell hypertrophy, by examining cardiac cell size and mRNA expression of hypertrophic cytokines. Under normal conditions, nm-aFGF had no impact on cardiac H9c2 cell shape and size. However, the cells were significantly enlarged under high glucose condition. In the cells exposed to high glucose with nm-aFGF, there was no hypertrophic effect. In addition, high glucose up-regulated mRNA expression of ANP and ANG in both H9c2 cells and rat myocytes, which were significantly attenuated by treatment with either native aFGF or nm-aFGF. These in vitro findings were confirmed by in vivo animal studies, e.g.: administration of nm-aFGF significantly prevented diabetes-induced ratio increase of heart weight to tibia length. Although there was a significant difference for the ratio of heart weight to tibia length only at 6 month, rather than at 1 month, the significantly increased mRNA expression of the molecular hypertrophic markers, ANP, ANG and β-MHC in the heart were observed at 1 month, suggesting the process of hypertrophy has started at molecular level from 1 month of diabetes. Treatment with nm-aFGF could protect diabetes-induced molecular levels of cardiac damage at the early-stage, resulting in a prevention of cardiac hypertrophy.

We also demonstrated the up-regulation of FN mRNA expression in the both in vitro cultured cells exposed to high glucose and in vivo diabetic mouse model at 6 month after diabetes onset. Using the in vitro model we also detected the mRNA expression of fibrosis markers FN and TGF-β. During the study we found high glucose significantly increased FN mRNA expression in H9c2 cells, HMVECs and rat myocytes, which was remarkably prevented by both native and modified aFGF treatment. The anti-fibrotic effect of nm-aFGF was equal to native aFGF. The in vitro finding was also confirmed during the in vivo study. Sirius red staining showed that at 6 months after diabetes onset there was a clear fibrotic response in the heart, which was significantly inhibited by nm-aFGF treatment. Moreover, administration of nm-aFGF to diabetic mice also prevented mRNA expression of fibrotic markers including FN and TGF-β1 in diabetic heart.

## Conclusions

In summary, diabetic cardiomyopathy is a clinical problem that develops in diabetes, and potentially involves oxidative stress and the subsequent cardiac myocytes death, hypertrophy and fibrosis. These pathogenic changes may contribute to compromised ventricular dysfunction in diabetes, which is the leading cause of death in the world today. In our present study, we found that nm-aFGF significantly protected cardiac cell death, hypertension, cardiac dysfunction and prevented cardiomyopathy in diabetic heart. By in vitro mechanistic study, we identified the protective mechanism of nm-aFGF against diabetic cardiomyopathy. We demonstrated that nm-aFGF has a similar cardiac protection to native aFGF, from diabetes. Considering that the modified nm-aFGF only losses its mitogenetic effect, but preserves all other bio-effects such as anti-apoptosis effect and cardio-protection [7], nm-aFGF maybe a potential candidate for the therapeutic application against diabetic cardiomyopathy in clinics due to its lack of oncogenic potential.
